# Supplementary material for: Rapid Identification of Brucella Genus and Species In Silico and On-Site Using Novel Probes with CRISPR/Cas12a
Source: Microorganisms. 2024 May 17;12(5):1018. doi: 10.3390/microorganisms12051018 (PMC11124060; doi:10.3390/microorganisms12051018)
Supplement: Supplementary file 1 [file microorganisms-12-01018-s001.zip › Supplemental File S1 Figures S1-S10.pdf]

Supplemental File S1

Figure. S1 The result of the deleted 11 *Brucella* strains by GAMOSCE V1.0 scanning.
Figure. S2 The result of the isolate (GeneBank: GCA_018604785.1) by GAMOSCE V1.0 scanning.
Figure. S3 The pre-experimental analysis of RPA oligonucleotide primers and crRNAs for BAPs.
Figure. S4 The pre-experimental analysis of RPA oligonucleotide primers and crRNAs for BMPs.
Figure. S5 The result of 104M, 2308 and two isolates via AMOS-PCR.
Figure. S6 The results of the Rose Bengal Test for serum in these blood samples.
Figure. S7 The genomes of blood samples extracted.
Figure. S8 These extracted genomes from blood samples were tested by AMOS-PCR.
Figure. S9 These extracted genomes from blood samples were tested by PCR with bscp31 target (227 bp).
Figure. S10 The sequencing result of RPA products from these positive blood samples.

***Brucella genus* specific fragments analysis.** To find the specific fragments for *Brucella genus*, we collected 25 loci including 5s rRNA(1), 16s rRNA, *omp22*(2) and *bscp31*. The most of them come from MLST (21 locus, https://pubmlst.org/bigsdb?db=pubmlst_brucella_seqdef&page=downloadAlleles&tree=1), which are *acnA*, *aroA*, *caiA*, *cobQ*, *csdB*, *ddlA*, *dnaK*, *fbaA*, *fumC*, *gap*, *glk*, *gyrB*, *int_hyp*, *leuA*, *mutL*, *mviM*, *omp25*, *prpE*, *putA*, *soxA*, *trpE*.

Screening specific eProbes of *Brucella* species. As previous research described(3), the chromosome of *B. melitensis* bv. 1 str. 16M strain (GenBank: GCA_000007125.1) as reference was *in silico* cut into 3,294,832 fragments (100-bp windows in 1-bp steps) using a python script. Using a local BLAST (BLAST-2.7.1+), we removed the fragments appearing in other *Brucella* species strains and that do not exist in any *B. melitensis* genomes. After elimination, there are still 73,142 fragments that appeared in all 353 *B. melitensis* genomes. These fragments were merged according to the overlaps between the fragments, and 1282 fragments for identifying *B. melitensis* were left (Fig. 1). The length of these fragments was between 100-bp and 369-bp. Based on the above fragments, 1084 fragments including the SNP site were uncovered with analysis of local BLAST and MEGA-X in all 762 *Brucella genus* genomes. The specific fragments of *B. abortus* and *B. ovis* were obtained by using the same strategy. Specific fragments containing SNPs are left, named eProbes.

Due to the extremely similar genomes of *B. suis* and *B. canis*, we established a query database with *Brucella suis* (GeneBank: GCA:000007125.1, renamed as Bsuis_001) as the reference sequence using the index command of bwa software in the Linux system. The mem command of bwa software is used to align the remaining genome sequences (n =772) to the reference sequence, and bcftools is employed to extract SNP sites between the genome sequence of each strain and the reference sequence. The matrix contain SNP site are processed using Python programming. Next, we screened 610 SNPs that could distinguish *B. suis* and *B. canis* from other *Brucella genus* strains. To obtain the specific fragments of *B. suis*, we further screened 214 SNPs that could distinguish *B. suis* from *B. canis*.

As the previous research described(4), taking the identified SNP in each locus as the base point, 50 bp upstream and 49 bp downstream of the SNP from Bsuis_001 genome was used to create a probe of 100 bp. These Probes were searched for among *Brucella genus* strains (773 strains) using a local BLAST software. After removed those non-specific probes, we obtained fourteen probes distinguishing *B. suis* and *B. canis* from other *Brucella genus* strains and fifteen probes distinguishing *B. suis* from *B. canis*.

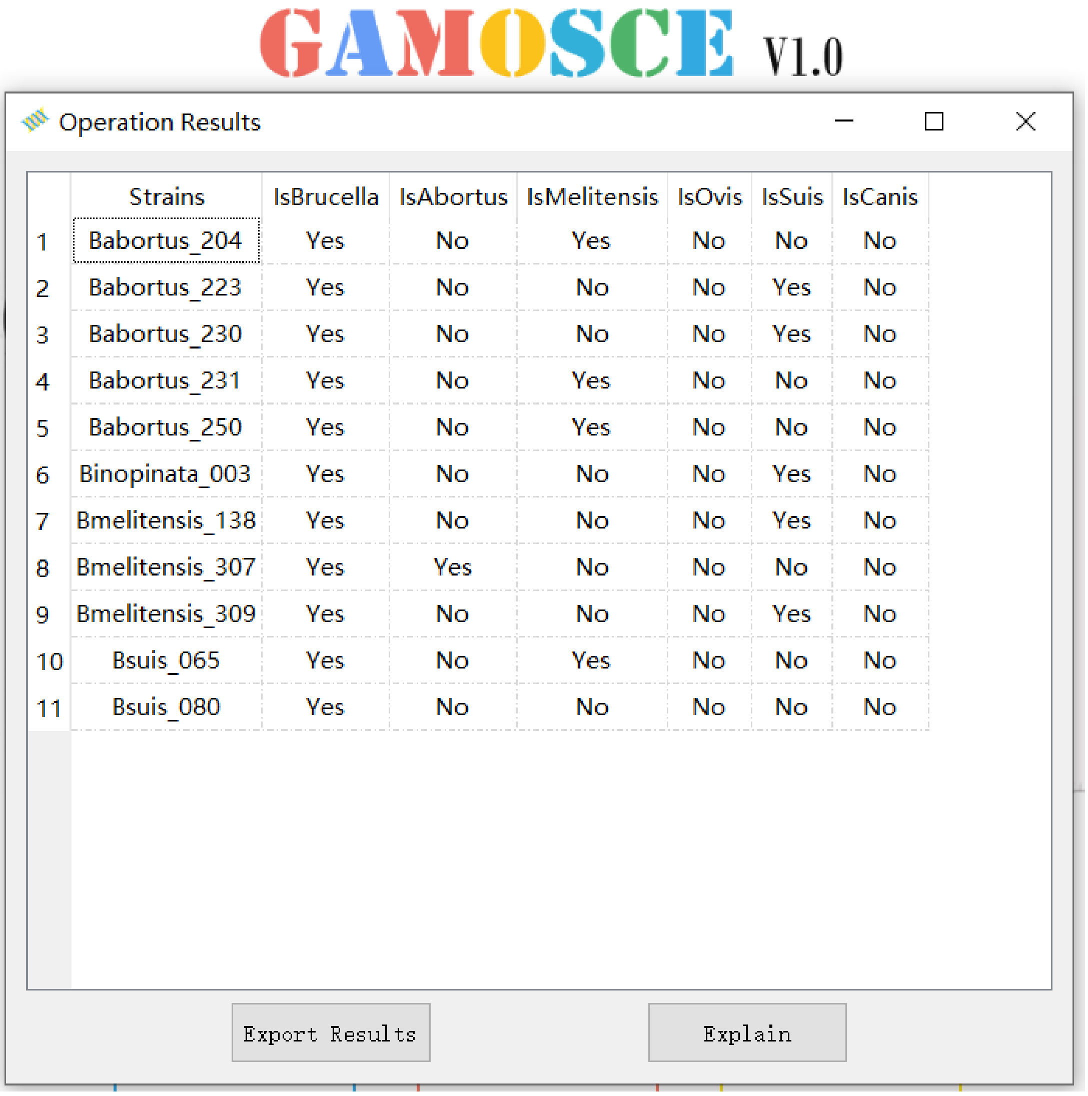

	Strains	IsBrucella	IsAbortus	IsMelitensis	IsOvis	IsSuis	IsCanis
1	Babortus_204	Yes	No	Yes	No	No	No
2	Babortus_223	Yes	No	No	No	Yes	No
3	Babortus_230	Yes	No	No	No	Yes	No
4	Babortus_231	Yes	No	Yes	No	No	No
5	Babortus_250	Yes	No	Yes	No	No	No
6	Binopinata_003	Yes	No	No	No	Yes	No
7	Bmelitensis_138	Yes	No	No	No	Yes	No
8	Bmelitensis_307	Yes	Yes	No	No	No	No
9	Bmelitensis_309	Yes	No	No	No	Yes	No
10	Bsuis_065	Yes	No	Yes	No	No	No
11	Bsuis_080	Yes	No	No	No	No	No

Figure S1. The result of the deleted 11 *Brucella* strains by GAMOSCE V1.0 scanning.

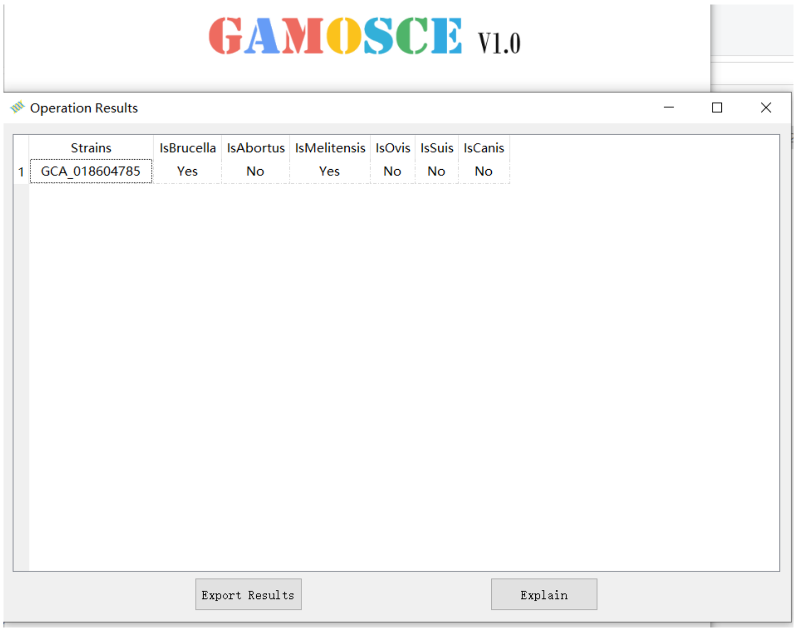

Figure S2. The result of the isolate (GeneBank: GCA_018604785.1) by GAMOSCE V1.0 scanning.

Detection of *B. melitensis* and *B. abortus* based on Cas12a-RPA. The Cas12a and RPA reaction were performed as previously described(3-5). The CRISPR/Cas12a system contained the Cas12a protein, the target DNA, specific CRISPR RNAs (crRNAs), and a single-stranded DNA (ssDNA) reporter. The Cas12a protein binds to the target DNA under the guidance of crRNA to form a Cas12a/crRNA/DNA complex. The complex can cleave single-stranded fluorescent DNA probes. The ssDNA (a 12-base probe) was labeled with 6-carboxyfluorescein (FAM) and the quenching group BHQ1: 5'-FAM-GAGACCGACCTG-3'-BHQ1. The strong green fluorescence could be seen with the naked eye under blue light in the wavelength range of 450 to 480 nm. Weak fluorescence appears when no target is encountered. The Cas12a reaction was conducted at 37 °C for 30 min in a 20-uL volume (Table S4). Fluorescence intensities were detected at 30 min using a Bio-Rad real-time PCR CFX96 instrument in FAM mode (Life Science, Hercules, CA, USA) or with the naked eye under blue light (Fig.1 and Fig. 4). The target DNA was provided by RPA amplification product. RPA reaction was performed following the manufacturer's protocol. The sequences of the chosen RPA oligonucleotide primers and crRNAs after pre-experimental analysis using RPA are shown in Table 1.

For the specific detection of *B. melitensis* or *B. abortus* genomic DNA, CRISPR RNAs (crRNAs) corresponding to the Probes of *B. melitensis* or *B. abortus* were designed and evaluated in the laboratory. SNP sites of probes are generally located at bases 1 to 6 downstream of the PAM sequence. The crRNA can target both the coding strand and the noncoding strand. The crRNAs for BAPs and BMPs were assessed by RPA-Cas12a with fluorescent signal (Fig. S3 and Fig. S4).

Lateral flow dipsticks (LFDs, #31203-01; ToloBio, China) were also used to display the detection results. Single-stranded 12-nucleotide DNA probes were modified with FITC and biotin sequences (FITC-5′-GAGACCGACCTG-3′-biotin) as described above. Lateral-flow-based immunochromatographic readouts rely on the high affinity of streptavidin and biotin and FITC binds

to gold-nanoparticle-labelled FITC-specific antibodies. When intact, the DNA probes remains bound to the streptavidin line ("C" line) through biotin, creating one color band on the test strip. When the DNA probes is cleaved, FITC and the bound gold-labelled FITC-specific antibodies flow farther on the strip and bind to secondary anti-species antibodies, which leads to the formation of a second color band ("T" line). The Cas12a reaction product (10 μL) with 40 μL NEB 3.0 buffer was placed in a PCR tube and incubated with an LFD strip (5 min). Cas12a reaction was conducted using a 20 pmol ssDNA probe.

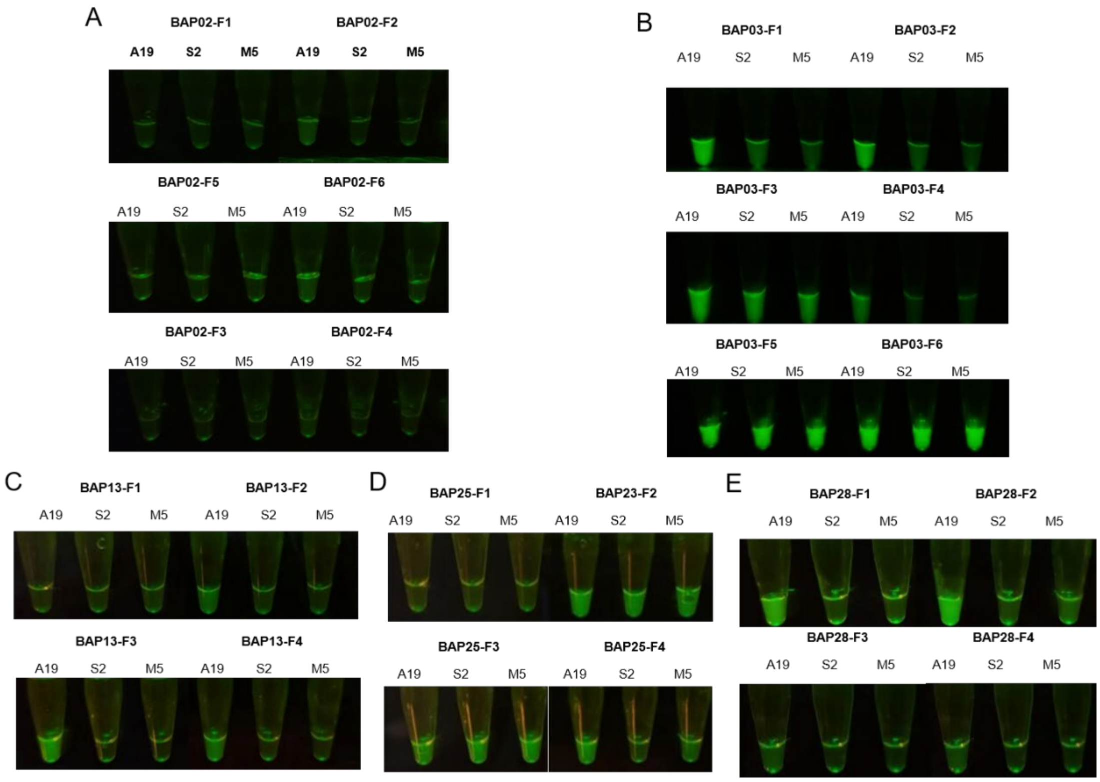

Figure S3. The pre-experimental analysis of RPA oligonucleotide primers and crRNAs for BAPs.

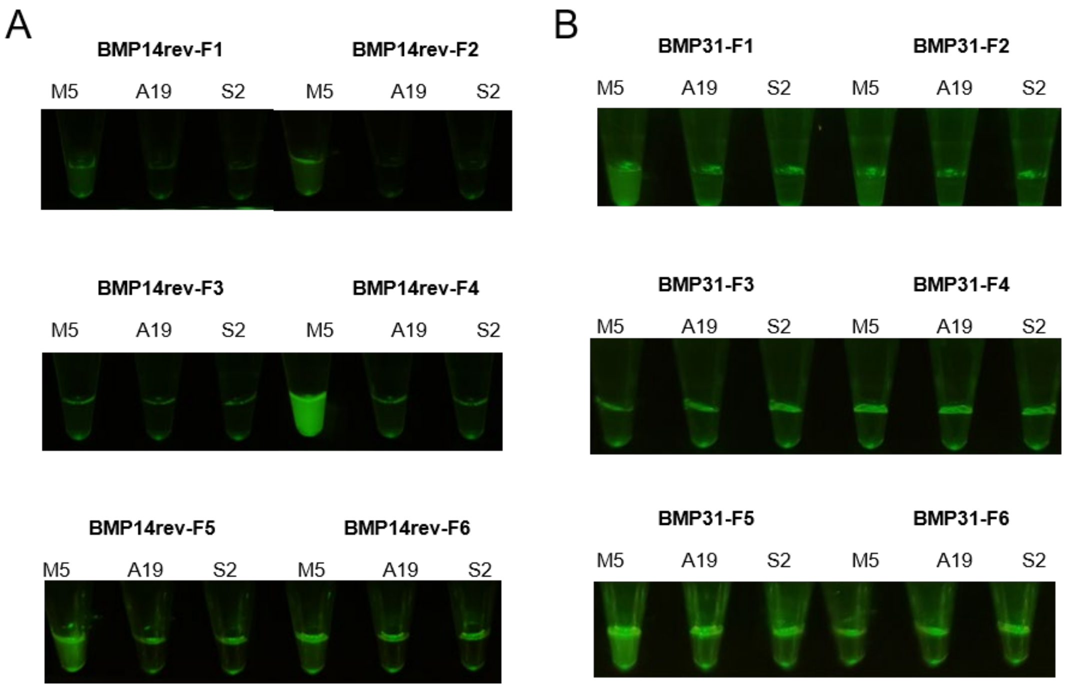

Figure S4. The pre-experimental analysis of RPA oligonucleotide primers and crRNAs for BMPs.

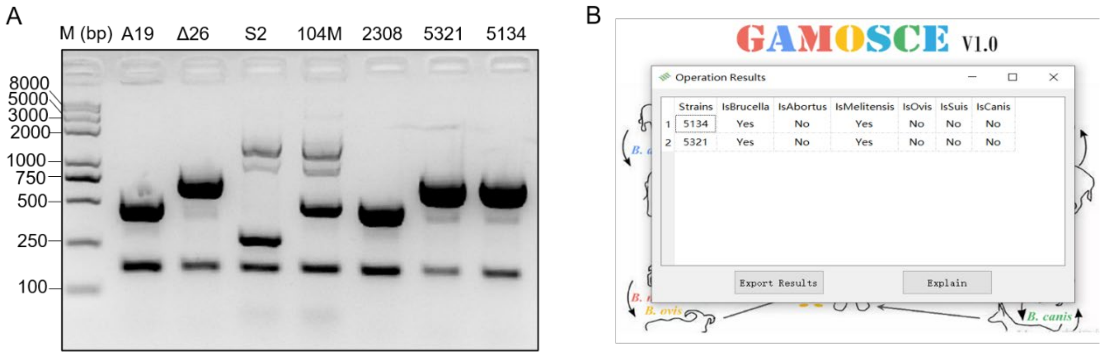

Figure S5. The result of 104M, 2308 and two isolates via AMOS-PCR.

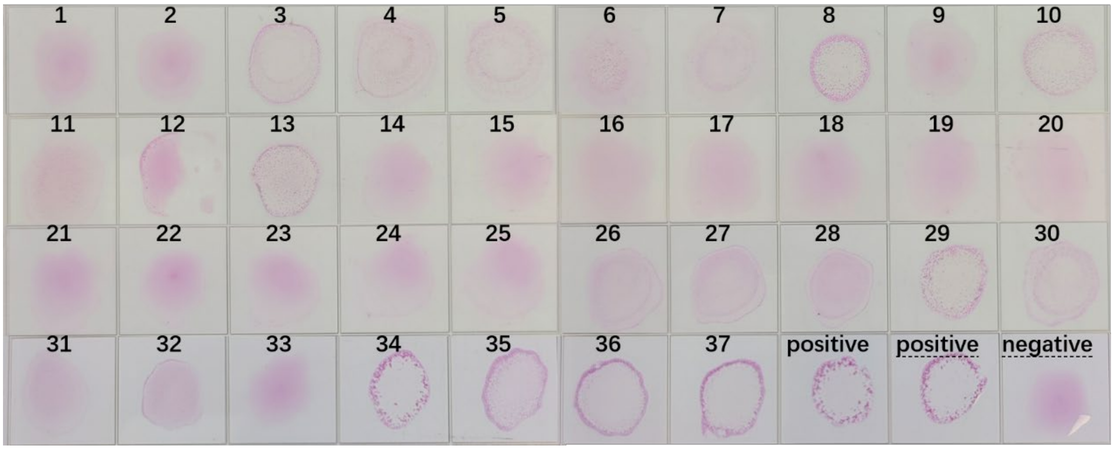

Figure S6. The results of the Rose Bengal Test for serum in these blood samples

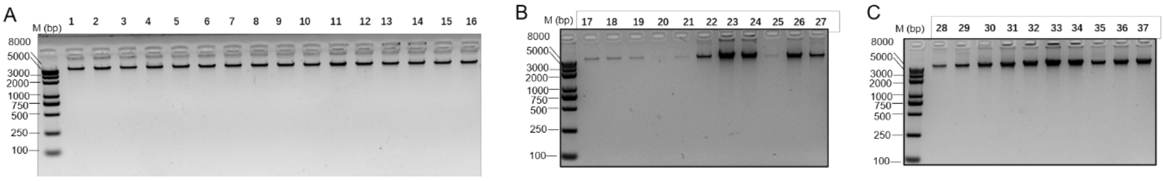

Figure S7. The genomes of blood samples extracted.

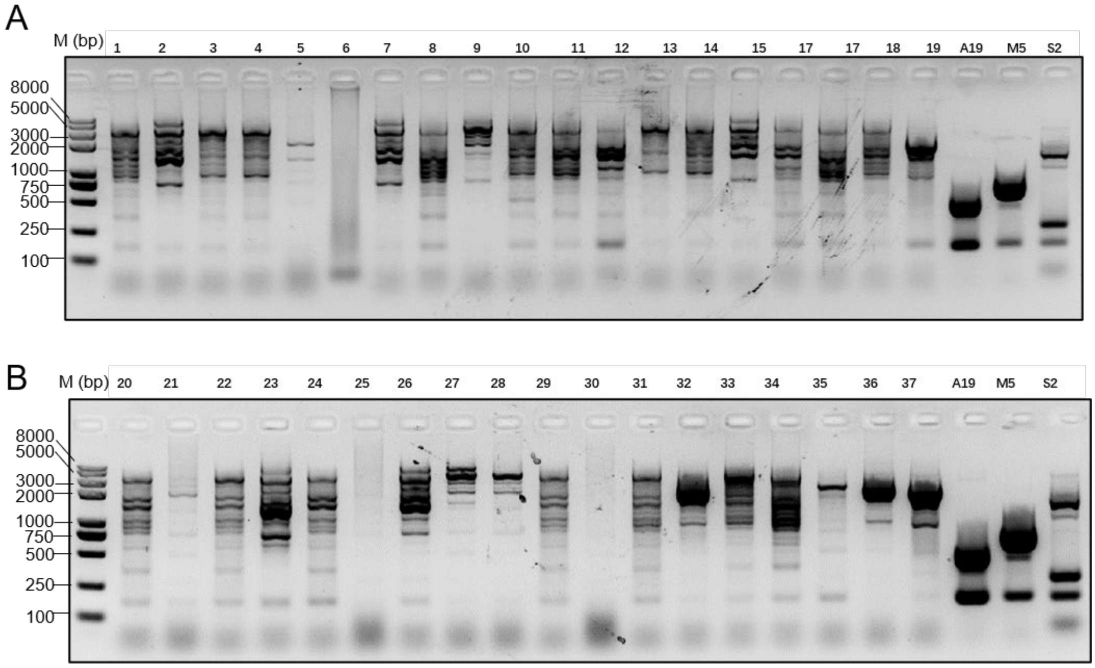

Figure S8. These extracted genomes from blood samples were tested by AMOS-PCR.

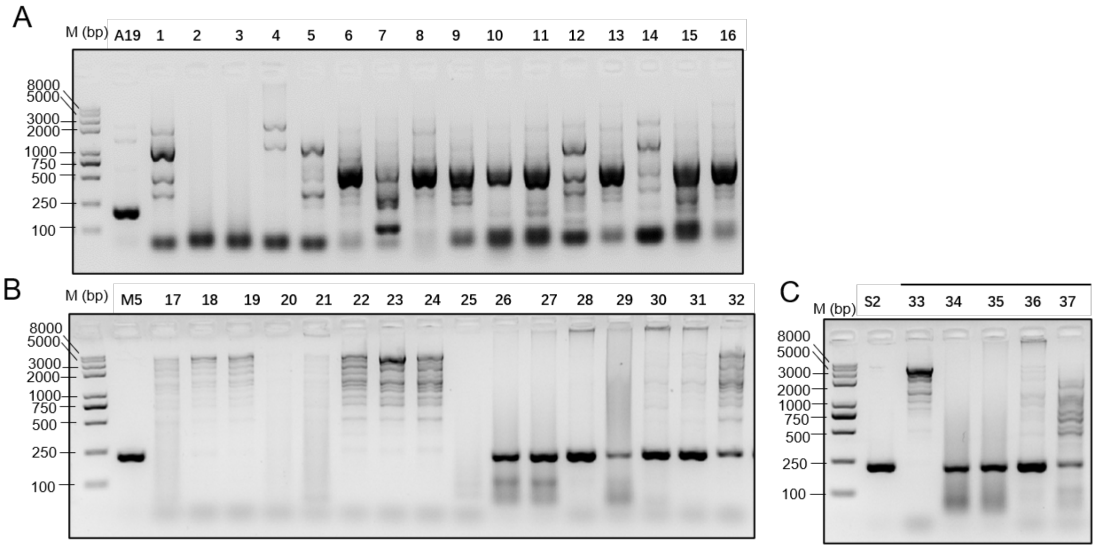

Figure S9. These extracted genomes from blood samples were tested by PCR with *bscp31* target (223 bp).

RPA products sequence of positive blood sample.

The BMP31 corresponding RPA products were sequenced as the Fig. S10 shown. The RPA products of *B. abortus* A19 and *B. suis* S2 at 83rd and 84th base is G and G as the blue lines indicated, but *B. melitensis* M5-90Δ26 is A and A. The result is consistent with the BMP31 of our BMPs set (Table S2b, marked in red.). The RPA products of other positive samples via RPA-Cas12a with BMP31 at 83rd also is A, completely consistent with M5-90Δ26. Their 84th base is A from the sequencing results of S34, S35, S36 and S37. These results provide support for the detection of clinical samples by RPA-Cas12a with BMP31.

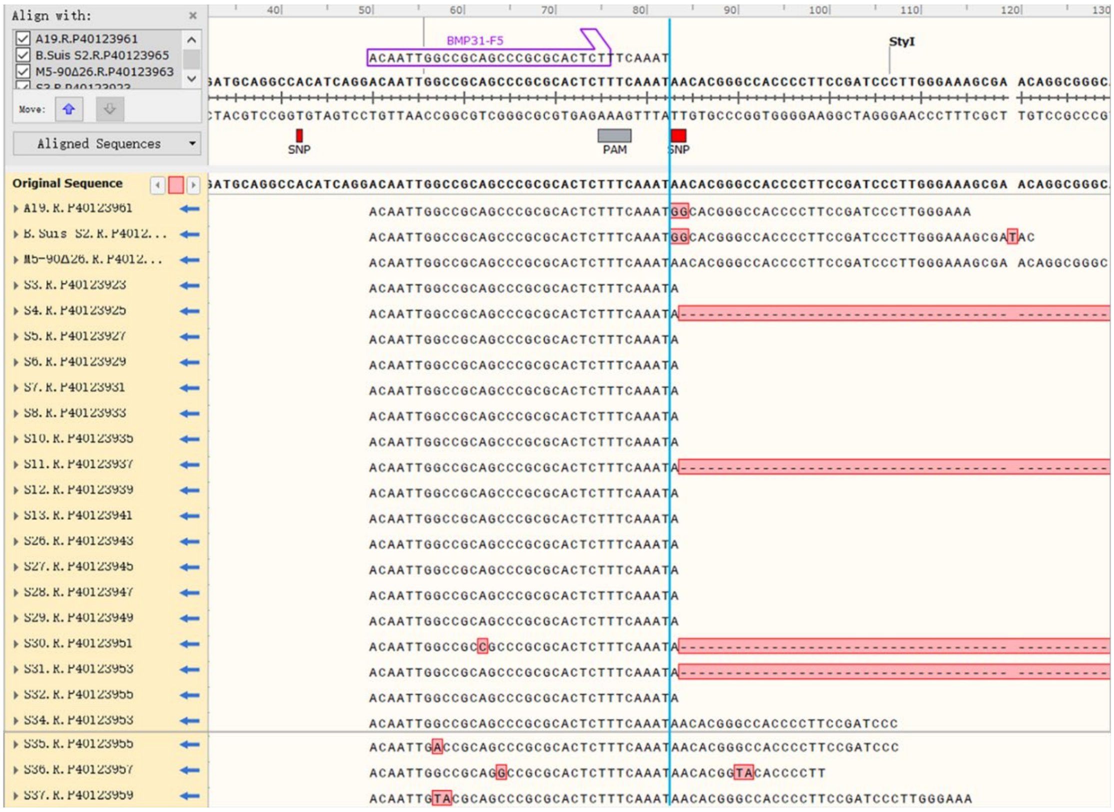

Figure S10. The sequencing result of RPA products from these positive blood samples.

REFERENCES

1. Minnick MF, Stiegler GL. 1993. Nucleotide sequence and comparison of the 5S ribosomal RNA genes of Rochalimaea henselae, R.quintana and Brucella abortus. Nucleic Acids Res 21:2518.
2. Wang Q, Zhao S, Wureli H, Xie S, Chen C, Wei Q, Cui B, Tu C, Wang Y. 2018. Brucella melitensis and B. abortus in eggs, larvae and engorged females of Dermacentor marginatus. Ticks Tick Borne Dis 9:1045-1048.
3. Chen G, Lyu Y, Wang D, Zhu L, Cao S, Pan C, Feng E, Zhang W, Liu X, Cui Y, Wang H. 2021. Obtaining Specific Sequence Tags for Yersinia pestis and Visually Detecting Them Using the CRISPR-Cas12a System. Pathogens 10.
4. Lyu Y, Wang D, Yuan L, Feng E, Zhu L, Pan C, Guo Y, Liu X, Wang H. 2022. Rapid Identification of Bacillus anthracis In Silico and On-Site Using Novel Single-Nucleotide Polymorphisms. Microbiol Spectr 10:e0228521.
5. Wang D, Chen G, Lyu Y, Feng E, Zhu L, Pan C, Zhang W, Liu X, Wang H. 2022. A CRISPR/Cas12a-based DNAzyme visualization system for rapid, non-electrically dependent detection of Bacillus anthracis. Emerg Microbes Infect 11:428-437.
