# Supplementary material for: Rapid Identification of Brucella Genus and Species In Silico and On-Site Using Novel Probes with CRISPR/Cas12a
Source: Microorganisms. 2024 May 17;12(5):1018. doi: 10.3390/microorganisms12051018 (PMC11124060; doi:10.3390/microorganisms12051018)
Supplement: Supplementary file 1 [file microorganisms-12-01018-s001.zip › Supplemental File S2 GAMOSCE user manual Figures S11-S14.pdf]

GAMOSCE user manual

Identify_Brucella
GAMOSCE V1.0
(Brucella Genus, Abortus, Melitensis, Ovis, Suis, Canis genome-based identification with E-probe)

This version is for Windows users, one-click identification of Brucella genus and 5 significant Brucella species. Users do not need to write code.

GAMOSCE is a tool based python that employs a combination of * in silico * the 126 specific tags of Brucella to rapidly identify *Brucella genus and 5 significant Brucella species * isolates using nucleotide sequencing data.

1. The software and test_data can be downloaded from http://github.com/844844/GAMOSCE for windows users. Click the code and then Download Zip (Figure S11).

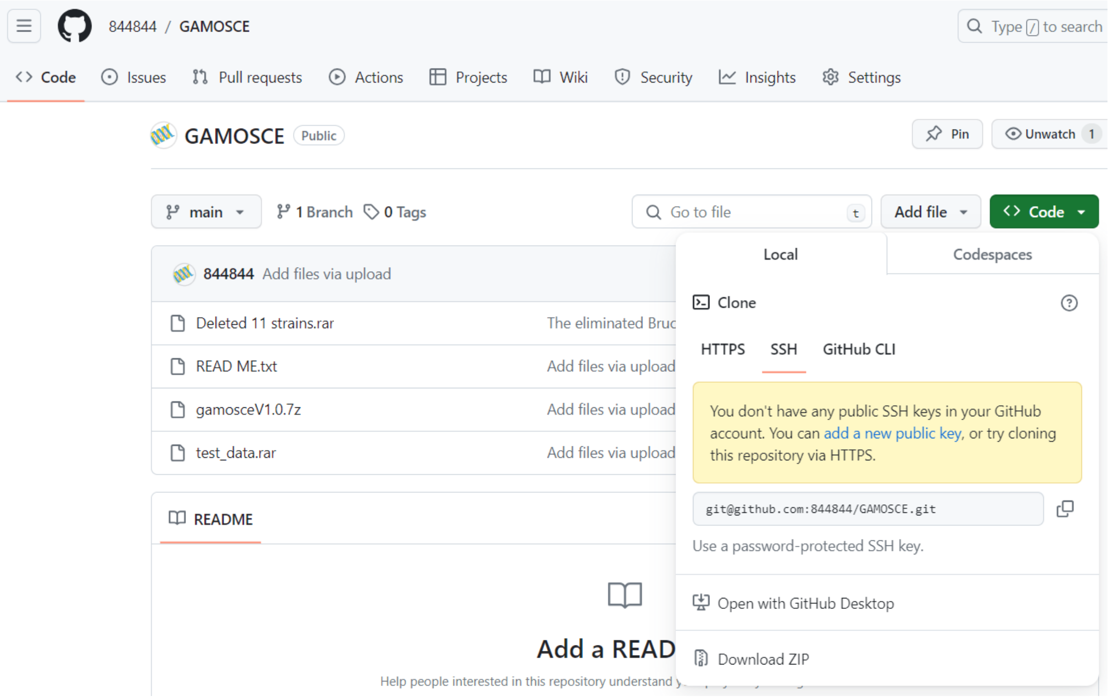

Figure S11. The website screenshot of Github.

2. Decompress GAMOSCE.zip, Open agile_start Shortcut (Figure S12).

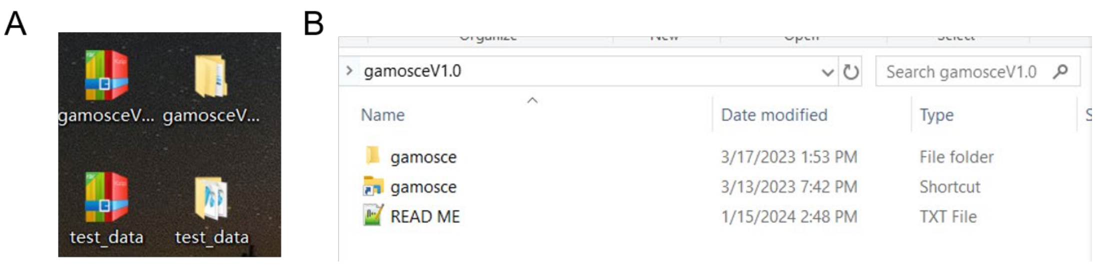

Figure S12. The website screenshot of decompressing.

3. In Figure S13, click Select File to open the sequencing file. After entering the sequencing results,

clicking open means start identification. Click on the result to export, output the result, and select export location and enter the file name, complete the analysis. Every column except the first in the result file is the identification result, whether it is *Brucella genus* or is *B. abortus*, *B. melitensis*, *B. suis, B. canis, B. ovis,* deterministic.

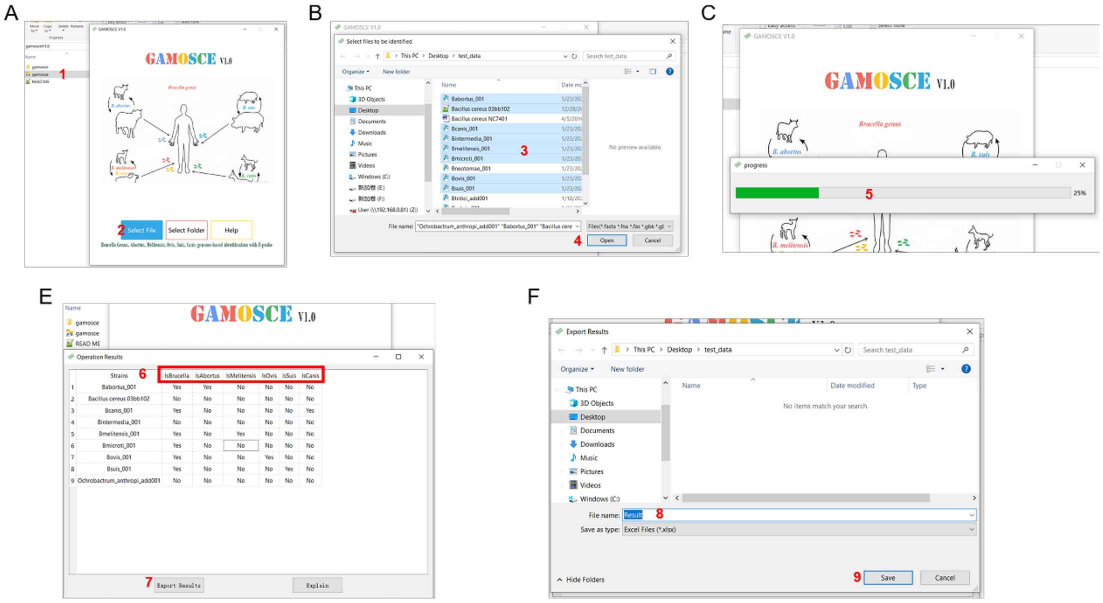

Figure S13. Workflow of the Select File model.

4. In Figure S14, click Select Folder to load sequence of whole folder. The common nucleic acid sequences can be loaded. Click on the result to export, output the result, and select export location and enter the file name, complete the analysis. The last column in the result file is the identification result, whether it is Ba or not Ba, deterministic.

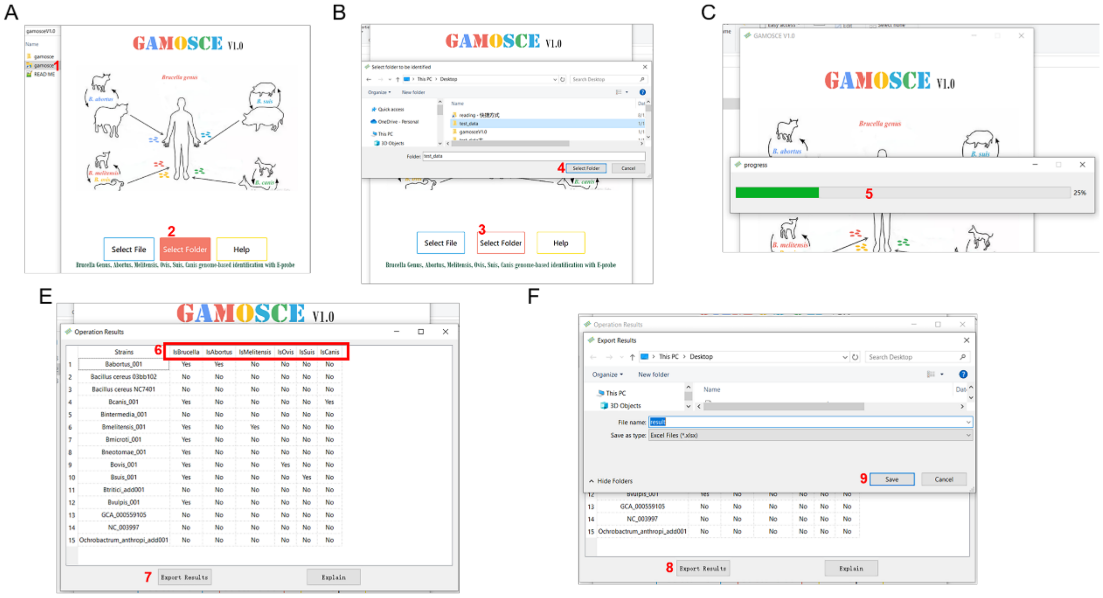

Figure S14. Workflow of the Select Folder model.

5. Note that office files in .doc, .xls, .ppt and other formats cannot be loaded. The identification result is determined based on whether the specific tags of *Brucella* are searched. The more tags are searched, the more reliable the target sample is positive.
